# Altruism and “love of neighbor” offer neuroanatomical protection against depression

**DOI:** 10.1016/j.pscychresns.2021.111326

**Published:** 2021-07-01

**Authors:** Lisa Miller, Priya Wickramaratne, Xuejun Hao, Clayton H. McClintock, Lifang Pan, Connie Svob, Myrna M. Weissman

**Affiliations:** a Spirituality Mind Body Institute, Clinical Psychology Program, Teachers College, Columbia University, New York City, USA; b Department of Psychiatry, Vagelos College of Physicians and Surgeons, Columbia University, New York City, USA; c Division of Translational Epidemiology, New York State Psychiatric Institute, New York City, USA; d Sierra Pacific MIRECC, San Francisco VA Health Care System, San Francisco, USA; e Department of Psychiatry and Behavioral Sciences, University of California San Francisco, San Francisco, USA; f Department of Epidemiology, Mailman School of Public Health, Columbia University, New York City, USA

**Keywords:** Spirituality, MRI, Altruism, Love of neighbour, Depression, High Risk

## Abstract

We prospectively investigate protective benefits against depression of cortical thickness across nine regions of a Ventral Frontotemporal Network (VFTN), previously associated with spiritual experience. Seventy-two participants at high and low risk for depression (Mean age 41 years; 22–63 years; 40 high risk, 32 low risk) were drawn from a three-generation, thirty-eight year study. FreeSurfer estimated cortical thickness over anatomical MRIs of the brain (Year 30) for each of the nine ROIs. Depression (MDD with SAD-L; symptoms with PHQ; Years 30 and 38) and spirituality (self-report on five phenotypes; Year 35), respectively, were associated with the weighted average of nine regions of interest. VFTN thickness was: 1) positively associated (p<0.01) with two of five spiritual phenotypes, altruism and love of neighbor, interconnectedness at a trend level, but neither commitment nor practice, 2) inversely associated with a diagnosis of MDD (SADS-L Year 30, for any MDD in the past ten years), and 3) prospectively neuroanatomically protective against depressive symptoms (PHQ-9 Year 38) for those at high familial risk.

## 1. Introduction

Human spirituality has been shown in previous research: 1) to be an innate capacity with 23–40% of the variance in adults attributed to heritability (Kendler et al., 1997a; 1999; Tsuang et al., 2002), following a developmental surge or “biological clock” of increased heritability across middle to late puberty (Koenig et al., 2008), 2) to have deep common expression around the globe, despite a diversity of religions and cultures, perhaps representing phenotypes of the heritable contribution (Benson et al., 2012; McClintock et al., 2016), and 3) to have clear and consistent neuroanatomical correlates seated in regions of perception, motivation, relationality (Beauregard and Paquette, 2006; Miller et al., 2014; Newberg, 2010; 2018). Spiritual capacity is expressed as a felt way of being, a set of experiences and perceptions, which for any given individual may or may not be wedded to a religious denomination, philosophy or theology.

Scientific work focused on human spirituality, by way of clarification, does not focus on the claims of religion or its tenets, and certainly stands distinct from any claims about the theological or cosmological nature of the world. Clinical science focuses on the human capacity for lived spirituality, experienced as perceptions, motivations and practices around transcendence and ultimate concerns. Often investigation focuses on the biological correlates and mental health consequences of personal spiritual life (Braam and Koenig, 2019; McClintock et al., 2019; Miller, 2012; Pargament and Mahoney, 2009; Piedmont et al., 2008; Piedmont, 2020).

A new growing area of work focuses on the neural correlates of spiritual experience (Miller et al., 2018; McClintock et al., 2019; Newberg, 2010; Newberg, 2012, 2018). To the best of our knowledge, to date the only MRI studies to examine the neural correlates of spirituality as directly protective against psychopathology were published by our team (Liu et al., 2017; Miller et al., 2014), showing in our longitudinal sample: (1) report of a high level of sustained spirituality to be associated with cortical thickness across regions of the parietal, precuneus and occipital, precisely where previous research had shown in recurrent depressives cortical thinness (Peterson et al., 2009), and (2) prospective protective benefits of relative cortical thickness in these regions against symptoms of depression, with the *greatest magnitude of benefit found in people at high familial risk for depression* due both to heritability and environmental stressors (Hao et al., 2017; Peterson et al., 2009; Weissman et al., 2016).

As the first published report to our knowledge to identify neural protective benefits of spirituality against depression, our study nonetheless was naturally conducted within the limitations of the field at the time. Only a hand-full of previous studies had identified the brain regions associated with spirituality at that time (Newberg, 2012; Urgesi et al., 2010), which formed our *a priori* targeted investigation around the regions of neural protection against depression. At the time of our original study, we assessed personal spirituality using the most frequently used single item measure, “How personally important is religion or spirituality to you?” While this item had been shown in the research literature to robustly predict medical outcomes, it confounds spirituality (which is an innate capacity) with religion (which is a socialized embrace of this capacity), and clearly is quite broad and non-specific.

Here, we advance our investigation of the neuroanatomical protective effect of spirituality against depression, given recent identification of: (1) universal spiritual phenotypes potentially reflecting the heritable contribution to spirituality, and (2) a distributed brain network including cortical regions that may be associated with the heritable dimension of spiritualty.

### 1.1. Phenotypes of spirituality

Evidence of a heritable or “hard-wired” component to spirituality suggests that cross-culturally spirituality may carry deep commonalities or phenotypes. In a study of over 5500 participants in India, China and the United States of highly diverse cultural and religious traditions, members of our team found the existence of at least five common phenotypes (McClintock et al., 2016), namely: Altruism, Love of Neighbor as Self, Sense of Oneness, Contemplative Practice, and Commitment to Religion/Spirituality. All five phenotypes held structural integrity across participants, with level of endorsement on each phenotype showing greater individual variance than group variance across these diverse countries and religions.

Of relevance for global mental health, common structural phenotypes were found across participants who represented the five most populous world religious traditions: Christianity 2.2 billion; Islam 1.5 billion; Non-Religious, “Spiritual but not Religious” and Secular 1.2 billion; Hinduism 1.1 billion; and Buddhism 535 million. In a subsequent study, turning to people at high and low risk for depression, we replicated the structural integrity of the five phenotypes as distinct from family risk for depression and past history of depression (McClintock et al., 2018), ruling out the argument of the tautological sameness of depression and spirituality as merely the absence of one another (Sloan et al., 1999). Evidence instead supports the five phenotypes both as being protective against depression and as being structurally independent from depression.

### 1.2. Neural correlates of the heritable capacity for spiritual experience

Ken Kendler and colleagues (Kendler et al., 1997a; 1999) using a genetic epidemiologic twin study originally identified a partially heritable contribution (29% of the variance heritable; 71% environmental) to a dimension of lived spirituality that authors termed *personal devotion*: a felt perception of a personal dynamic relationship with G-d, Jesus, Hashem, Allah, the Higher Power, the Universe, or whatever personal term might be used to describe a loving guiding and protective ultimate reality; in brief, a transcendent relationship. A personal transcendent relationship has been found to be protective against depression across a number of studies and various assessment measures (Braam and Koenig, 2019; Miller, 2012;Pargament et al., 2004).

To explore the potential neural correlates of the perception of the transcendent relationship, a collaborative team of fMRI investigators adapted an established in-scanner task, previously found to be valid across other forms of personal experience (such as craving, fear or depression) (Miller et al., 2018). In the adapted task, participants first received an individual interview to elicit palpable, detail driven memory of lived spiritual connection with G-d or the Higher Power. The detailed retelling of the memory was scripted, recorded and then played back in-scanner to participants, a method previously shown across fifteen years of research to elicit the neural and physiological correlates of living the experience itself (plus those of memory) (Sinha, 2013). The spiritual in-scanner task was conducted within an fMRIstudy to identify the engagement of a Ventral Frontotemporal Network (VFTN) (McClintock et al., 2019b). Engagement of the VFTN during personal spiritual experience was found across culturally and religiously diverse young adults (18–26 years), including those to report being spiritual but not religious. The VFTN appears to be a neural seat of perception of a transcendent relationship across religious traditions and a range of expressions of spirituality.

The findings of the fMRI study were interpreted to suggest that the VFTN likely buffers against stress and trauma (McClintock et al., 2019a), although the direct potential protective benefits were not directly tested. This VFTN, engaged during a felt perception of the transcendent relationship, is comprised of middle and inferior frontal cortices, frontal opercula, and anterior insula. Regions, when activated, are associated with shifts in attentional patterns and a felt relationality with fellow human beings and/or a Higher Power.

### 1.3. The current study

In keeping with our ongoing line of inquiry on the neural correlates of spiritual experience and related protective benefits, we now investigate the possibility that people with a strong personal spirituality show *structural* neuroanatomical differences across the VFTN, as measured by relative cortical thickness across the constituent regions. In this exploratory study, we further assess if this potential network wide anatomical difference might be associated with a diagnosis of Major Depressive Disorder (MDD; current or prospective) and/or protect against severity level of depressive symptoms (current or prospective).

We turn in this moment to a high risk sample, marked by elevated rates of depression, stress and negative life events (Weissman et al., 2016)(Weissman, 2021), to investigate a pathway of neuroanatomical protection against depression based upon spiritual phenotypes. The current investigation benefits from a 38-year inter-generational longitudinal study yielding adults at high and low risk for depression. Specifically, structural MRI on adult offspring at high and low familial risk for depression allows us to ask: (1) might greater cortical thickness across the regions of the VFTN be associated with any or each of the five phenotypes? and (2) might cortical thickness across the VFTN be protective against depression, either diagnosis of MDD or level of symptoms of depression, particularly in people at high risk for depression?

## 2. Methods

### 2.1. Participants

The study at each wave was reviewed and approved by the New York State Psychiatric Institute Institutional Review Board. Informed written consent was obtained for adults for themselves and for their minor children, verbal assent was also obtained from minors.

Participants were part of a 38-year longitudinal familial study of 72 adults (age 22 63 years) who were the 2nd or 3rd generation participants of depressed or non-depressed probands. While an extensive description of the methodology of the longitudinal study has been published (Weissman et al., 2016)(Weissman, 2021), of relevance to the current study is that the first generation of study participants was recruited from a Depression Clinic in New Haven Connecticut, together with demographically matched controls from the same geographic area. Using the criteria from a structured diagnostic interview, the probands met criteria for MDD, while matched controls did not meet diagnostic criteria for MDD. The MDD diagnosis of first generation participants defined familial risk status for all subsequent generations, as either high risk or low risk for depression.

For the current study, we acquired MRI scans on 114 participants at Time 30 from the overall study. There were no inclusion or exclusion criteria for participation in the MRI study beyond those of the overall longitudinal study. One participant was removed from analysis due to severe head motion during scan. Twenty-seven of the remaining 113 participants scanned using MRI at Year 30 did not complete spirituality measures at Year 35. Out of the 86 participants with both Year 30 MRI and Year 35 spirituality measures, 14 of them were younger than 18 years when scanned and, thus, were removed from the study due to age. This resulted in 72 participants for the current analysis (40 high risk and 32 low risk). Of the current study sample, 39% of the participants were male, and mean age of the participants was 41.4 years (See Table 1 for demographics and depression status of participants).

There were no significant differences between the participants in the high and low risk groups with respect to overall level of report of spirituality on any of the five phenotypes. Participants in the high risk group, at both Time 30 and Time 38, had higher rates of a Schedule for Affective Disorders and Schizophrenia–Lifetime (SADS-L) diagnosis of MDD (42.5%; 62.5%) than did participants in the low risk group (12.5%; 22%). High risk participants were slightly older than participants in the low risk group (46.1 years versus 39.4 years). All analyses were controlled for age and gender.

Participants had whole brain magnetic resonance imaging (MRI) scans in Year 30. Regions of interest (ROIs) were placed on the MRI scans and their cortical thickness and corresponding surface areas were estimated using FreeSurfer. In addition, participants were assessed for a diagnosis of MDD using the SADS-L and level of severity of depressive symptoms using the PHQ-9 at Year 30 and again at Year 38. Participants were assessed for the five spirituality factors at Year 35, due to the timing (and related support) of the overall longitudinal study. Although MRI was not again run in year 35, a high level of stability of cortical thickness between Year 30 and Year 35 can be inferred from published research on this longitudinal study sample showing stability of cortical thickness across *eight years* in people at high and low risk for depression (Hao et al., 2017).

### 2.2. MRI scanning

MRI scans were obtained using a GE Signa 3T whole-body scanner (GE Medical Systems, Milwaukee, WI) equipped with an 8-channel, phased array head coil using three-dimensional fast spoiled gradient recall sequence (repetition time = 4.7 ms, echo time = 1.3 ms, 110° flip angle, bandwidth = 41.67 MHz, field of view = 25 × 25 cm, array spatial sensitivity encoding technique factor = 2, 1 mm slice thickness, 256 × 3 × 256 matrix size, 128 slices, 0.98 × 0.98 × 1.0 mm voxel resolution, 1 NEX images × 2).

ROIs were placed on the MRI scans and their cortical thickness and corresponding surface areas were estimated using FreeSurfer (Fischl, 2012). The nine regions spanning the VFTN, specifically the caudalmiddlefrontal, entorhinal, fusiform, inferiortemporal, medialorbitofrontal, parsopercularis, rostralmiddlefrontal, superiortemporal and insula, were identified using the Desikan–Killiany atlas (Desikan et al., 2006). The posterior cingulate cortex and inferior parietal lobe were excluded, as they show negative activation, rather than positive activation, in the functional study identifying the VFTN. Transposing the functional network to a set of structural, neuroanatomical regions using the Desikan–Killiany atlas generated a series of structural, nine ROIs, which, taken together, were comprehensive but slightly broader than the VFTN, posing a slightly increased challenge to a statistical signal. Mean cortical thickness values for each of these ROIs were generated using standard procedures from FreeSurfer. The mean cortical thickness of each of the nine ROIs then was weighted by its area. The weighted values were summed to create an overall mean thickness of the nine ROIs for the left and right hemispheres as consideration of each hemisphere allows for the possibility of asymmetry in the findings.

### 2.3. Measures

Anatomical MRIs of the brain were acquired (T1-weighted images) and we used Freesurfer (Desikan et al., 2006) to estimate cortical thickness at each point on the pial surface to provide ROIs thickness and its corresponding surface area of the cortex.

Diagnosis of MDD was made via a diagnostic interview, using the SADS-L adult version (Manuzza et al., 1986). The SADS-L is a semi-structured interview administered individually by a trained clinical researcher from which can be derived a number of DSM diagnoses. Of relevance to the current study is diagnosis of MDD. The SADS-L allows for lifetime or current diagnosis of MDD, episode of MDD since previous assessment, and severity of lifetime or current episodes. Of relevance to the current MRI study was use the SADS-L to generate follow-up assessments at two time points: (1) Year 30 for a follow-up diagnosis of an episode of MDD over the past ten years, since the time of the last assessment (Year 20–30), and (2) Year 38 for a follow-up diagnosis of an episode of MDD over the eight years since the last assessment (Year 30–38). The categorical diagnosis of MDD (SADS-L) is binary (positive or negative for an episode MDD during this time period). Any statement with regard to diagnosis of MDD in this current study was generated using the SADS-L.

Current level of depressive symptoms was assessed using the Patient Health Questionnaire (PHQ-9) (Kroenke and Spitzer, 2002). The PHQ-9 is a 9-item self-report measure designed to assess severity of level of symptoms, from which can be assigned a diagnostic category of depression using cut-off scores. Participants are asked to rate how often in the past two weeks they have been bothered by depressive symptomatology (e.g., “Little interest or pleasure in doing things?” and “Feeling down, depressed, or hopeless?”) and vegetative symptoms (e.g., “Feeling tired or having little energy”). Participants rate the severity of symptoms using a 4-point Likert scale ranging from 0 (*Not at all*) to 3 (*Nearly every day*). Research on the PHQ-9 suggests the screener is sensitive to level of depressive symptoms with clinical cut-offs as follows: 0–4 no depression, 5–9 mild, 10–14 moderate, 15–19 moderately-severe and 20–27 severe. In the current study, the PHQ-9 is used exclusively to assess the level of depressive symptoms at Year 30 and Year 38, with all related analyses using the scale continuously (rather than categorically by level of depressive symptoms).

The five spiritual phenotype factor scores were based upon response to nine self-report measures, which previously had been shown to replicate the five phenotype factors in people at high and low risk for depression (McClintock et al., 2018). Factor scores were assigned based upon the weight of the item loadings along each of the five factors. Specifically, the following nine instruments were used: (a) the Intrinsic Religiosity subscale of the Duke University Religion Index (Koenig and Büssing, 2010); (b) the Belief Salience Scale (Blaine and Crocker, 1995); (c) Compassion Scale as modified by Krause and Hayward (Krause and Hayward, 2015); (d) sitting and moving contemplation items assessed whether or not participants had regularly engaged in such practices (McClintock et al., 2016); (e) the Spirituality in Nature scale (Drapkin et al., 2016); (f) the ontological, psychological, and social subscales within the Sorokin Multidimensional Index of Love Experience (Levin and Kaplan, 2010); (g) the Eco-awareness subscale of the Spirituality Scale (Delaney, 2005); (h) the universality subscale of the Spiritual Transcendence Scale (Piedmont, 1999); and (i) the humanistic engagement subscale of the SpREUK-P Questionnaire (Büssing et al., 2005). As described by McClintock et al. (2018), a weighted least square means and variance adjusted (WLSRV) estimator was employed using oblique quartimin rotation for five factors. Factor scores were then determined based on the regression method of factor score estimation (Skrondal and Laake, 2001).

The five phenotypes correspond to items such as these: *Altruism:* I work voluntarily for others. When I see someone in a difficult situation, I try to imagine how they feel. I help others. *Love of Neighbor as Self:* Love for love’s sake brings the greatest happiness. The best kind of love is given freely. For a friend in need, I would sacrifice almost anything. Without having others to love, life wouldn’t be worth living. *Interconnectedness:* Spending time in nature helps me to feel more connected spiritually. At times, I feel at one with the universe. I believe there is a larger plan to life. I believe there is a connection between all things that I cannot see but can sense. *Contemplative Practice:* On average, how often do you practice meditation, in terms of days per month? How many months have you been doing mind-body practices? *Commitment to Religion/Spirituality:* My religious beliefs are what lie behind my whole approach to life. Being a religious person is important to me. I participate in religious events (i.e., religious congregations etc.). I try hard to carry my religion over into all other dealings in life.

### 2.4. Analysis

We examined the association between the overall average thickness of the 9 regions of the VFTN and relative degree of risk for MDD (SADS-L) at Year 38, controlling for diagnosis of MDD at Year 30, participant’s age, gender and risk status, for the left and right hemispheres separately, using regression analyses with Year 38 diagnosis of MDD as the outcome and the weighted average of the nine regions as the predictor.

We then repeated the analysis focusing on the level of severity of depressive symptoms using the PHQ-9. Here we examined the association between the overall average thickness of the 9 regions of the VFTN and level of depressive symptoms reported on the PHQ-9 at Year 38, controlling for report on the PHQ-9 at Year 30, participant’s age, gender and risk status, for the left and right hemispheres separately. Using regression analyses with Year 38 level of severity of depressive symptoms on the PHQ-9 as the outcome and the weighted average of the nine regions as the predictor.

The sample was then stratified by risk status and both sets of analyses were repeated, consistent with the statistical methodology of our previous findings on cortical thickness and depression (Miller et al., 2014).

In addition, we examined the associations between the network predictor and each of the five spiritual phenotype factors (McClintock et al., 2016) by means of a series of regression model with each of the five factor scores, in turn, as the outcome, and the average cortical thickness of the regions that comprise the network as the independent variable, while controlling for diagnosis of MDD as measured by the SADS-L at Year 30 and the potential confounders of age, gender and risk status. Exploratory analysis was also conducted by stratifying the sample by risk status.

We then examined the association between each of the five spirituality factors, in turn, with a diagnosis of MDD as measured by the SADS-L at Year 38 (any episode of MDD between Year 30-Year 38), controlling for diagnosis of MDD as measured by the SADS-L at Year 30. Finally, analyses were repeated using the PHQ-9 for depression, as an assessment of level of severity of depressive symptoms. We examined the association between each of the five spirituality factors, in turn, with a report on the PHQ-9 at Year 38, controlling for report on the PHQ-9 at Year 30.

## 3. Results

Demographics and clinical status of the sample are presented in Table 1 by high and low risk group. A higher rate of diagnosis of MDD as measured by the SADS-L at baseline for the current study (Year 30) and at follow-up (Year 38) were found in the high risk group as compared to the low risk group.

Table 2 shows mean *z*-scores along each of the five spiritual phenotypes by high and low risk group. While only significant at the level of a trend, overall lower levels of report of Altruism, Love of Other as Self, and Commitment to Religion/Spirituality were found in the high risk as compared to low risk group.

### 3.1. Cortical thickness by risk status

**Fig. 1.**
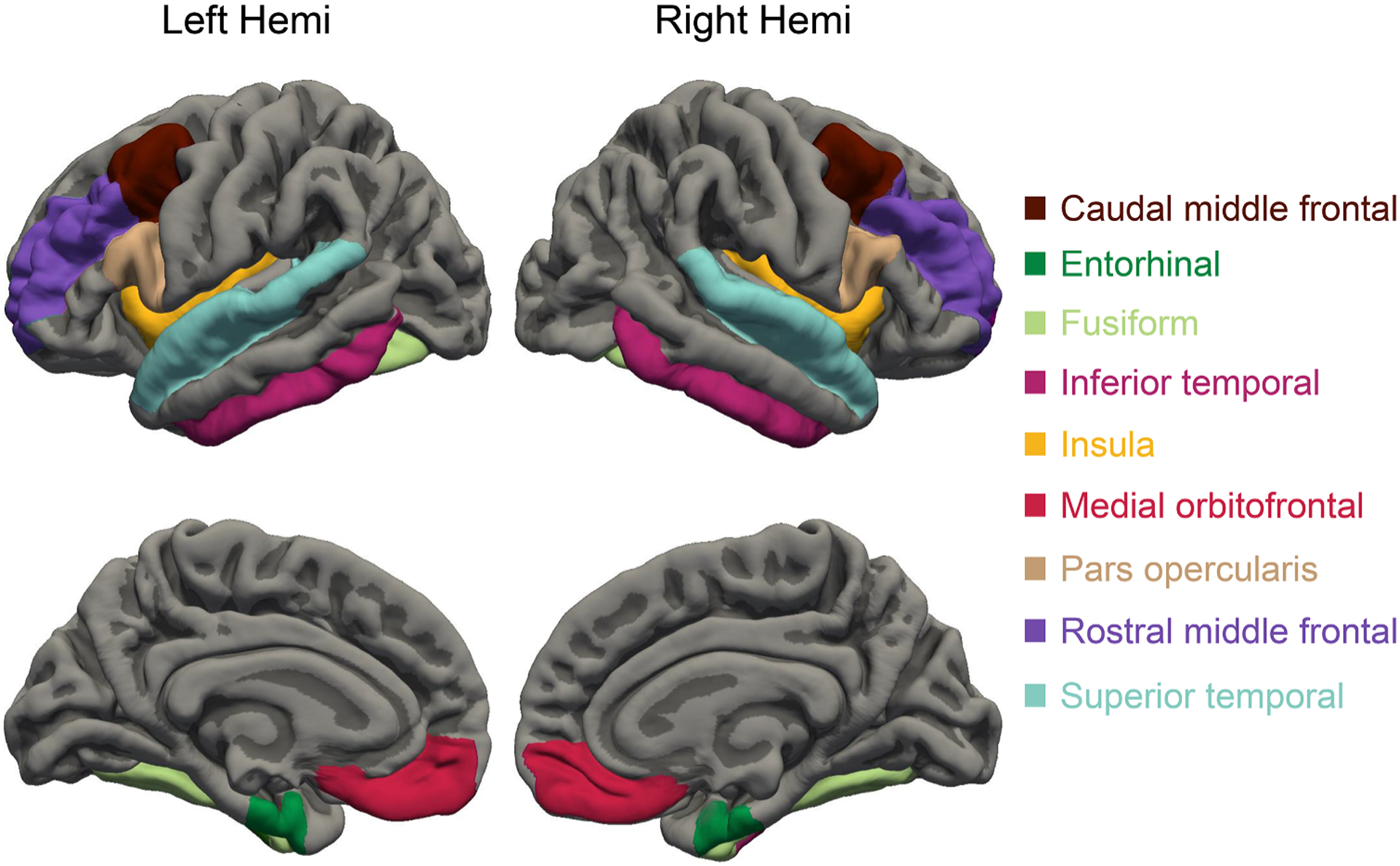
VFTN cortical region of interest (ROI).

Fig. 1 illustrates the nine cortical regions across the VFTN. Overall, as compared with the high risk group, the low risk group showed relatively greater average cortical thickness across the regions of the VFTN in both the left hemisphere and the right hemisphere: LH: LRMean = 2.72 (0.09), HRMean = 2.65 (0.12), *t* = 2.61, *p* = 0.011; RH: LRMean 2.73 = (0.12), HRMean = 2.66, *t* = 2.17, *p* = 0.33.

At the level of a statistical trend and at a small magnitude, greater cortical thickness was found in the right hemisphere as compared to the left hemisphere of the VFTN, in the full sample (RHMean = 2.69 (0.12), LHMean = 2.68 (0.11), *p* = 0.055) and in the high risk group (RHMean = 2.66 (0.13), LHMean = 2.65 (0.12), *p* = 0.083) but not in the low risk group. This small right-left asymmetry in cortical thickness is consistent with that asymmetry previously published from a large and diverse international sample (*N* = 17, 141) of healthy adults (Kong et al., 2018) wherein there is no related clinical manifestation and it is considered “normal.” Whereas all regression analyses in the current study to include cortical thickness control for age, mean cortical thickness when compared between groups at high and low risk for depression may be confounded with age.

### 3.2. Average cortical thickness of the nine regions and depression as measured by the SADS-L and PHQ-9

Results of regression models to determine the relationship between the average cortical thickness of the VFTN and depression (both diagnosis of MDD on the SADS-L and level of severity of depressive symptoms on the PHQ-9) by familial risk status for depression showed differential findings by risk status. Of note is that much overlap exists between level on depressive symptoms as measured by the PHQ and diagnosis of MDD on the SADS-L, with the PHQ more sensitive to change for being used as a continuous measure (not categorical) with greater variance in this study.

Greater average cortical thickness was robustly and significantly associated with decreased relative risk for diagnosis of MDD using the SADS-L (Year 30) in both left and right hemispheres among the high risk sample: LH (OR = −11.323, *p* = 0.012); RH (OR = −7.149, *p*= 0.047). Findings were not significant among the low risk sample. While controlling for this association of baseline MDD (Year 30), average cortical thickness was not significantly associated with Year 38 MDD, most likely due to the 98% overlap between diagnosis of MDD at Year 30 and Year 38.

Increasing average cortical thickness was *prospectively* associated with decreasing level of symptoms of depression as measured by the PHQ-9 in both left and right hemispheres in the high risk sample: LH (*b* = −0.427, *t* = −2.44, *p* = 0.02); RH (*b* = −0.314, *t* = −1.88, *p* = 0.069), while controlling for baseline PHQ-9 (Year 30).

At the level of a trend, increasing average cortical thickness was associated with increasing symptom of depression as measured by the PHQ-9 in both hemispheres in the low risk group LH (*b* = 0.246, *t* = 1.750, *p*= 0.092), RH (*b* = 0.281, *t* = 1.960, *p* = 0.061) controlling for Year 30 PHQ-9, suggesting differential pathway of resilience following previous elevated depressive symptomology by risk group.

Because the prospective findings point in opposite direction by risk status, overall in the full sample, there were no significant associations between cortical thickness and PHQ-9 symptoms in the overall sample in either the left or right hemisphere. LH (*b* = −0.140, *t* = −1.260, *p* = 0.213); RH (*b* = −0.060, *t* = −0.540, *p* = 0.593).

### 3.3. Cortical thickness & five spiritual phenotypes

We used linear regression models to establish the relationship between average cortical thickness of the VFTN and each of the spiritual phenotypes. Among these five phenotypes, uniquely, Altruism and Love of Neighbor as Self showed a strong and statistically significant positive association. That is, increasing cortical thickness was associated with increasing scores of the respective phenotypes in both the left and right hemispheres: Altruism: LH: (*b* = 0.342, *t* = 2.460, *p* = 0.017), RH (*b* = 0.331, *t* = 2.480, *p* = 0.016); Love: LH (*b* = 0.303, *t* = 2.220, *p*= 0.03); RH (*b* = 0.36, *t*= 2.79, *p* = 0.007). In short, greater cortical thickness across the VFTN was associated with greater Altruism and Love of Neighbor as Self. The magnitude of the positive association between average cortical thickness and level of self-report on phenotype, was similar for the two phenotypes of Altruism and Love of Neighbor as Self.

Neither Contemplative Practice nor Commitment to Religion/Spirituality showed a significant association with average cortical thickness across the VFTN. The lack of association was found across both hemispheres and both high and low risk samples. The differential association of cortical thickness across the VFTN by phenotypes suggests that these associations (Altruism and Love of Neighbor) were not artifactual of overall greater cortical thickness in depressed and non-depressed participants.

There were no significant differences between high and low risk samples.

There was evidence for a positive association between average cortical thickness and Sense of Interconnectedness scores, but findings were inconsistent when potential confounders of age, gender, risk status and Year 30 diagnosis of MDD were simultaneously included in the regression model, probably due to insufficient statistical power.

### 3.4. Prospective association between spirituality factors and depression (MDD and PHQ-9)

*Diagnosis of MDD*. Results of logistic regression models to determine the association between each of the five spirituality factors and a Year 38 diagnosis of MDD using the SADS-L showed that in the overall sample, after controlling for age, sex, risk status and *baseline diagnosis MDD (Year 30),* only Altruism was found to have a statistically significant *prospective* association with a diagnosis of MDD over ten years. Increasing Altruism scores were associated with decreasing relative risk of an episode of MDD (OR = 0.322, *p*= 0.019) as assessed by the SADS-L over the period of eight years between Year 30 and Year 38. There were no significant differences in the pattern of this association between high and low risk samples.

Love of Neighbor as Self scores at Time 35 were inversely associated with a diagnosis of MDD at Year 38 (OR = 0.319, *p* = 0.077) using the SADS-L at the level of a statistical trend, with increasing Love of Neighbor Score associated with decreased risk of developing an episode of MDD between Year 30 and Year 38, uniquely among people at high risk for depression.

### 3.5. Symptom severity level

Results of linear regression models, where PHQ-9 scores were regressed on each spirituality factor, in turn, to establish the relationships between these spirituality factors and level of severity of depression symptoms as measured by the PHQ-9, showed that, after controlling for age, gender risk status and *baseline* PHQ-9 scores at Year 30, in the overall sample, only Love of Neighbor as Self was significantly related to PHQ-9 scores at Year 38. Increasing scores for Love of Neighbor as Self were *inversely* related to level of severity of depressive symptoms as measured by the PHQ-9 at Year 38, (*b* = −1.01, *t* = −2.02, *p* = 0.048). When high and low risk samples were examined separately, it was found that this pattern was restricted to the high risk sample (*b* = −1.955, *t* = −3.06, *p* = 0.005); there was no significant association in the low risk sample (*b* = 0.285, *t* = 0.350, *p* = 0.732).

## 4. Discussion

Neuroanatomical protection against depression was found in adults at high familial risk for depression who had a relatively stronger sense of Altruism and Love of Neighbor as Self. The neuroanatomical protective benefits were not seen in people at low risk for depression, nor found for other dimensions of lived spirituality that we studied (such as report of Contemplative Practice or Commitment to Religion/Spirituality). Specifically, our findings reveal: (1) *altruism* and *love of neighbor* to be associated with greater cortical thickness across the network (VFTN) previously associated with the in-scanner task of recalled personal spiritual experience, specifically a felt transcendent relationship with the Higher Power, G-d, the Universe (or a related personal term), (2) greater cortical thickness across the VFTN to be inversely associated with a diagnosis of MDD over the past ten years and prospectively protective against level of severity of depressive symptoms eight years later, specifically in people at high familial risk for depression, and (3) altruism to be prospectively protective against diagnosis of MDD and Love of Neighbor to be prospectively protective against level of severity of depressive symptoms.

### 4.1. VFTN & relational spirituality

Altruism and Love of Neighbor correlate with cortical thickness across the regions of the VFTN, a network previously shown to be engaged in perception of the transcendent relationship, drawing on regions of bonding and enhanced perception. Findings of this study show that the neural correlates of a transcendent relationship are shared with a sense of care for fellow people. Here we see potential ***common ground of neuro-perception*** between, on the one hand, a sense of ***feeling love and support from G-d*** (or Higher Power), and on the other hand, ***feeling love and supporting fellow people***. These neuroanatomical findings may suggest that altruism and love of neighbor are experienced not exclusively as interpersonal events but via spiritual perceptions and motivations. A spiritual sense of human-human relationality within previous psychological research has been conceptualized as *relational spirituality*, leading to greater respect for dignity, acceptance, forgiveness, commitment and unconditional love. From the view of relational spirituality, fellow human beings are seen and felt as sacred, holding the presence of G-d or emanations created by the Higher Power (Mahoney, 2013)(Mahoney, 2010; Pinsent, 2013; (Tomlinson, 2016). To this body of research on relational spirituality, the current report offers a potential ***neuroanatomical common ground*** between human-human relationships felt as sacred and the transcendent-human relationship felt with G-d or the Higher Power.

### 4.2. VFTN and depression

In people at high risk for depression, cortical thickness across the VFTN was associated with decreased relative risk for MDD over the past ten years (at Year 30), and, prospectively, a decreased level of severity of depressive symptoms (at Year 38).

Can sustained practice of relational spirituality, as altruism and love of neighbor, augment cortical thickness in the spiritual network? The current study does not directly address change in cortical thickness associated with lived altruism and love, due to the study design. However, other studies suggest that: (1) a sustained form of contemplative practice or mindfulness (Newberg, 2012)(Holzel et al., 2011)(Yang et al., 2019)( ), and (2) spiritual practice taken together with positive social engagement (Newberg, 2010)(Newberg, 2018)( ), are associated with morphological change and, in some people, synaptogenesis. Taken together the convergent evidence is grounds for future research that might examine morphological change associated with sustained relational spirituality in the forms of altruism and love of neighbor. The implications of a *relational spirituality* intervention, focusing on altruism and love, offers great hope as a learned pathway to resilience against depression in people at high risk.

Uniquely among people at high risk for depression, altruism and love of neighbor offer *prospective* neuroanatomical protection against depressive symptoms. The neuroanatomical protective effects of relational spirituality are particularly striking in light of: (1) the overall relatively lower rates of altruism and love of neighbor in the high risk group (as compared to the low risk group) at the level of a trend and (2) overall relative cortical thinness across the VFTN in people in the high risks group (as compared with the low risk group). The neural impact of relational spirituality on depression appears greatest for those who otherwise most suffer.

The findings show that people at high risk struggle with interpersonal dimensions of spiritual life *per se* (but not the other dimensions of Contemplative Practice, Sense of Oneness, or Commitment to Religion/Spirituality) perhaps as part of broader challenges in social functioning (Barton et al., 2013) or due to relational history (Sandage et al., 2011) (Reinert, 2014). Consistent with the current findings, depressive social struggles (severing of relationship, avoidance or hostility) may sometimes involve an attenuated sense of relational spirituality, as has been demonstrated clinically (Cusi et al., 2011; Donges et al., 2005; Kupferberg et al., 2016). Conversely, improvements in relational spirituality may augment social functioning, given the common neural underpinnings around social perception and motivation.

Several overlapping pathways may yield the neuroanatomical benefits of altruism and love. From the view of social support, altruism draws people out of isolation into reconnection, beyond a sense of un-worthiness to valuable contribution, affecting both those who are helped and the helper (Neugebauer et al., 2019). Neuro-perception of fellow humans as spiritual beings or sacred relations may motivate service, forgiveness, and acceptance – all shown to be uplifting of the kind-hearted seer and doer and helpful in recovery from depression (Kasen et al., 2014). Service to others deepens one’s inner spiritual life, as a form of spiritual practice, care for creation, or worship in action, yielding greater spiritual understanding, which in turn brings an added degree of resilience against depression (Musick et al., 2003; Pargament et al., 2009; Sandage et al., 2011; Svob et al., 2019). Finally, a direct protective effect may exist between greater cortical thickness across regions associated with relational spirituality and decreased risk for depression. Here a loving way of being, feeling and living may be potentiated by a neural seat of awareness uniting the transcendent with embodied care.

### 4.3. Limitations

Limitations may include the smaller sample size. However, we emphasize that (1) due to the uniqueness of a high and low risk inter-generational sample, the statistical signal is strong, and (2) adequate sample size for longitudinal neuroimaging studies generally has been established as 30 participants, which is less than half the size of our study sample (Suh et al., 2019). Those regions identified by Desikan–Killiany Atlas, were broader than the VFTN shown in the previous fMRI study, such that as compared to the previous study, ours would seem to yield a weakened signal. Yet, the current neuroanatomical study identified a set of clear, significant findings. Relative cortical thickness across the VFTN might be seen as merely a global index of a “good brain.” However, the data from our study demonstrate that cortical thickness across the VFTN is uniquely associated with the relational aspects of spirituality, but not aspects of Contemplative Practice or Commitment to Religion/Spirituality.

The larger longitudinal study assessed for MDD using the SADS-L and PHQ-9 at Time 30 and Time 38, but not at Time 35, the timing of the spirituality assessment. However, among those participants positive for a diagnosis of MDD at Year 30, 98% were the same at both Year 30 and Year 38; the possibility of, at most, two unidentified cases of MDD at Time 38 strengthen the findings. MRI scans were conducted at Year 30, not concomitant with the spirituality measures at Year 35. The MRI findings are quite unlikely to have changed between Year 30 and Year 35, as previous research on the sample from the larger longitudinal study shows high stability of cortical thickness across *eight years* in people with high and low familial risk for depression (Hao et al., 2017).

Finally, the current study asks whether or not cortical thickness across the VFTN, at the *level of a network*, might prevent against depression and correlate with specific phenotypes of spiritual life. Although beyond the scope of the current study, future research might consider the unique contribution of each of the constituent regions, by investigating: (1) the relative protective benefits against depression of the cortical thickness of each region, and (2) the association of the cortical thickness of each region, with level of spirituality, for each phenotype. That said, our method applies a novel approach by examining structural brain differences associated with spirituality both: (1) across an entire network, and (2) along multiple dimensions of spiritual life, an investigative approach specifically to have been called for from within the field (Van Elk, 2019).

### 4.4. Implications

Depression is the leading cause of disability affecting 264 million people around the world (World Health Organization, 2020). This study reveals an innate human capacity for resilience, healing and renewal brought from an inborn spiritual neural capacity. Our innate neural endowment for altruism and love offers a pathway to prevent depression, particularly for those at high risk. Findings offer a potential pathway for augmented treatment and prevention: altruistic service, delivered with love.

Altruism and Love of Neighbor as Self might be cultivated by patients and families and supported, as part of treatment, by clergy, physicians, and mental health professionals. Widely used treatments such as Cognitive Behavior Therapy (CBT) or Interpersonal Psychotherapy (IPT) might integrate homework that initiates acts of service and practices of relational love, both as visualization (such as envisioning new acts of relational love, and, heart centered mediations designed to send out love to fellow human beings) and in action (such as planned engagement with family or in volunteer service, and, spontaneous acts of kindness). As well the therapist-patient alliance might be engaged as a form of relational spirituality, creating a powerful, sacred form of relationality perhaps never before experienced by the patient. In this way, the alliance may: (1) cultivate a felt sense of relational spirituality that the patient might bring forward to people outside of the session, and (2) bolster the neural capacity that also is a seat of awareness for a relationship with the Higher Power and transcendent awareness in daily life. The converse process of relational expansion might be true as well: patients might be encouraged to engage the Higher Power or transcendent relationship (in their own language and on their own terms) as a source of guidance to re-envision and strengthen interpersonal relationships.

To date, a host of spiritually oriented treatments (Richards and Bergin, 2005, 204; Shafranske and Sperry, 2005; Walker et al., 2015) have been published, with some approaches being applicable across faith traditions or apart from any faith tradition, while other spiritually oriented treatment models are grounded within a specific faith tradition. Standard assessment of each patient’s spiritual worldview and related perspective on suffering is integrated into intake as part of the SPIRIT program at Harvard Medical School, McLean Hospital and then leads to treatment planning for both inpatient and outpatient settings (Rosmarin et al., 2019). Aten and Leach (2009) have published a thorough resource for spiritually integrated treatment at each phase in the arc of care that integrates both transcendent practice and relational spiritual connections. Sandage et al. (2020) have published extensively on models of relational spirituality (RSM) *per se* as the foundational frame and focus of treatment.

Relatively few attempts represented in the clinical literature have been made to foster spiritual awareness as it extends to relational spirituality; effecting the ***dual seat of spiritual perception*** identified in this study. Designed as an intervention to “jump start” the VFTN, Awakened Awareness (AA) is a foundationally spiritual intervention (Miller, 2005; L. 2011)(Miller, 2011) to have been selected by the American Psychological Association as part of the “master” training series. AA aims to enhance spiritual perception through a method that is inclusive of members of any religious tradition, as well as those who identify as spiritual but not religious (SBNR) and humanists (who find spirituality in relationships with fellow human beings). Based upon twenty years of development in a secular and diverse setting, the foundational AA model is explicitly spiritually oriented, has been delivered in private and public organizations, treatment settings and to teachers and school administrators, the US Military, mental health and addiction settings and recently has been tested as a prevention model on college campuses to address the current epidemic of depression and despair in young adults (Scalora et al., 2021). AA offers *in vivo* practices to engage the “perceptual building blocks” of the VFTN to include inner reflection and visualization, outer observation on the confluence of life events, and dyadic practices of relational spirituality to deepen relationships. Relationships with people are considered to be spiritual events, meriting reflection and appreciation. Building of spiritual perceptual capacity often helps: (1) to awaken participants to an expanded sense of the self as connected with one’s true highest self, other people’s highest selves, and one’s own sense of the Higher Power or Transcendent Presence, (2) to heighten daily awareness of the confluence between “inner life” and “outer life,” (3) to emphasize the inherent spiritual nature of relationships or relational spirituality, and (4) to cultivate spiritual awareness as a guide in decision making. In its arc of delivery, AA draws on six progressive phases: (1) validity of internal awareness of experience, (2) witness and appreciation of whole self and whole other, (3) synchronicity and alignments in living, (4) building transcendent capacity through guided spiritual visualization and practices, (5) sharpening observation by noting and journaling on witness of a sacred, loving and guiding universe in daily life, and (6) creating a dialectical stance of living in the universe;seeing that humans do not control but rather interact with the flow of life, and, that meaning is not so much built but revealed through living.

Broad clinical implications of the findings from this study might include consideration around the make-up of the treatment team for addressing depression. Some patients with a strong personal spirituality may prefer that treatment is delivered by a spiritually oriented psychotherapist. Other patients might prefer treatment to be delivered through a partnership between a psychotherapist and a member of the clergy of their own faith tradition. These two models might be viewed as forming a continuum of care for uniting spirituality and psychotherapy in treatment (spiritually-oriented psychotherapy versus a partnership model), that might be made explicit by psychotherapists so that a patient is able to make an informed treatment decision.

### 4.5. Conclusion

In sum, for people at high risk for depression, the neural underpinnings of *relational spirituality* (Musick et al., 2003; Svob et al., 2019), Altruism and Love of Neighbor as Self, were inversely associated with a diagnosis of MDD over the past ten years and were, prospectively, protective against severity of depressive symptoms over the ensuing eight years.

Altruism *per se* prospectively protects against a diagnosis of MDD. Love of Neighbor as Self, prospectively, decreases severity of symptoms in those at high risk for depression. Taken with a body of research that shows altruism creates healing and renewal (Neugebauer et al., 2019; Pargament et al., 2009; Sandage et al., 2011; Svob et al., 2019), the current study reveals a foundational neuroanatomical underpinning to the protective effect against depression among people with vulnerability towards mood disorder.

Beholding fellow humans as spiritual beings to be loved and helped is neuroanatomically protective against depression in the *good-doer*. Whereas previous research has focused on the beneficial psycho–social sequela of altruism (for instance, a sense of purpose or personal value; other people kindly reciprocate), to the best of our knowledge this is the first published study to show the protective benefit of altruism against depression at the level of brain morphology. Altruism and Love of Neighbor are associated with cortical thickness across regions in the brain engaged during transcendent forms of perception of the Higher Power (G-d, Universe, Divine Presence or whatever a person’s related name may be). This neural concomitance potentially implies that altruism and love of neighbor engage the same perceptual “lens” for viewing sacredness in fellow people as used to perceive the Higher Power; a way of seeing and feeling *relational spirituality* in daily life that protects against depression, quite possibly in those people most at risk.

Cortical thickness across the VFTN may represent robust development of the perceptual capacity previously identified in twin-study research, the variance of which is explained roughly one-third by broad heritability and two-thirds by the environment (Kendler et al., 1997; 1999) and which has been shown to protect against depression. Relative strength of development of the innate neural capacity across the VFTN may represent a healthier and more resilient cultivation of brain and psyche. Humans may be “hard-wired” for mental health through care of fellow human beings.

“Whoever saves a single life saves the world entire.” (Talmud, Sanhedrin 37a) often read to reflect a moral imperative, may also reveal a pathway to human wholeness.

## Figures and Tables

**Table 1:** Demographics and major depressions by high and low risk groups.

Characteristics		Full sample (*N* = 72)	High risk (*N* = 40)	Low risk (*N*=32)	chisq^a^	*P*-value
Age at year 30	Mean(SD)	41.43(12.67)	44.43(11.98)	37.66(12.68)	−2.32	0.023
	Range	(22.04, 63.14)	(22.94, 63.14)	(22.04, 61.23)		
Age at year 38^b^	Mean(SD)	43.13(12.79)	46.12(12.06)	39.47(12.90)	−2.21	0.031
	Range	(23.85, 65.21)	(24.38, 65.21)	(23.85, 63.89)		
Gender	Female	44(61.11)	26(65)	18(56.25)	0.57	0.449
	Male	28(38.89)	14(35)	14(43.75)		
Generation	2nd	42(58.33)	29(72.5)	13(40.63)	7.43	0.006
	3rd	30(41.67)	11(27.5)	19(59.38)		
Major depression year 30 (Follow-up year 20–30)	Yes	21(29.17)	17(42.5)	4(12.5)	7.74	0.005
	No	51(70.83)	23(57.5)	28(87.5)		
Major depression^c^ Year 38 (Follow-up year 30–38)	Yes	17(25.76)	11(30.56)	6(20)	0.95	0.329
	No	49(74.24)	25(69.44)	24(80)		

a For age at wave 6.5 and wave 7, t-statistic presented in this column and Pr>|*t*| in the next column.

b There are 2 missing values in high risk group and one in low risk.

c There are 4 missing values in high risk group and 2 in low risk.

**Table 2:** Means scores of the five phenotypes^d^ by high and low risk groups.

Factor	Mean scores	Full Sample (*N* = 66)	High risk (*N* = 36)	Low risk (*N* = 30)	*t* statisticst	Pr > |*t*|
Altruism	Mean(SD)	−0.086(0.896)	−0.211(0.919)	0.064(0.859)	1.25	0.217
	Range	(−2.753, 1.713)	(−1.831, 1.713)	(−2.753, 1.491)		
Love	Mean(SD)	−0.144(0.885)	−0.274(0.934)	0.013(0.812)	1.32	0.192
	Range	(−2.166, 1.806)	(−2.166, 1.806)	(−1.376, 1.428)		
Commitment	Mean(SD)	−0.258(0.959)	−0.404(0.980)	−0.083(0.919)	1.36	0.178
	Range	(−1.887, 1.628)	(−1.887, 1.044)	(−1.724, 1.628)		
Contemplation	Mean(SD)	0.853(0.297)	0.885(0.333)	0.814(0.249)	−0.97	0.338
	Range	(0.307, 1.745)	(0.628, 1.745)	(0.307, 1.599)		
Interconnectedness	Mean(SD)	0.018(0.895)	0.041(0.977)	−0.010(0.800)	−0.23	0.820
	Range	(−2.326, 1.879)	(−2.326, 1.879)	(−1.598, 1.314)		

d There are 4 missing values in high risk group and 2 in low risk.
